# Understanding HPV Vaccine Uptake Among American Indian and Alaska Native College Students: A Scoping Review

**DOI:** 10.3390/ijerph23081027

**Published:** 2026-08-06

**Authors:** Vanessa Wright, Katy Fisher-Cunningham, Brayton Amidon, Shari Clifton

**Affiliations:** 1 Fran and Earl Ziegler College of Nursing, University of Oklahoma Health Campus, Oklahoma City, OK 73117, USA; katy-fishercunningham@ou.edu (K.F.-C.); shari-clifton@ou.edu (S.C.); 2 Texas A&M Health Science Center, Bryan, TX 77807, USA; bamidon@tamu.edu

**Keywords:** American Indian, human papillomavirus, HPV, vaccines, college

## Abstract

**Highlights:**

**Public health relevance—How does this work relate to a public health issue?**

- AI/AN populations experience disproportionate rates of HPV-associated cancers, making HPV vaccination a critical cancer prevention strategy.
- AI/AN college students ages 18–26 are eligible for catch-up vaccination but remain an understudied population with unique factors influencing vaccine uptake.

**Public health significance—Why is this work of significance to public health?**

- This review identified only three eligible publications, all derived from a single study conducted in 2009, demonstrating a substantial lack of contemporary evidence regarding HPV vaccination among AI/AN college students.
- Available evidence identified low HPV knowledge, stigma, healthcare mistrust, limited perceived risk, and access barriers as factors influencing vaccine uptake.

**Public health implications—What are the key implications or messages for practitioners, policy makers and/or researchers in public health?**

- Culturally grounded, community-engaged HPV vaccination initiatives are needed to improve vaccine uptake and reduce HPV-associated cancer disparities among AI/AN emerging adults.
- Improved collection and reporting of disaggregated AI/AN data are needed to inform public health policy, practice, and research.

**Abstract:**

American Indian and Alaska Native (AI/AN) communities experience disproportionate rates of HPV-associated cancers, yet vaccination uptake remains low. This scoping review mapped research on HPV vaccination among AI/AN college students aged 18–26, including vaccine uptake, knowledge, perceptions, and barriers. Using Joanna Briggs Institute scoping review methodology and the PRISMA-ScR checklist, MEDLINE (Ovid), CINAHL Ultimate, Embase (Ovid), ERIC (Ovid), Scopus, and Web of Science Core Collection were searched with no restrictions on publication year. Eligible studies included AI/AN college students aged 18–26 and examined factors influencing HPV vaccine uptake; studies that did not disaggregate AI/AN data were excluded. Of 911 abstracts identified, three publications met inclusion criteria; all included studies drew exclusively on American Indian samples and did not include Alaska Native participants. The findings reveal low HPV knowledge, limited perceived risk, gender differences in attitudes, and barriers including stigma, access challenges, and mistrust of healthcare systems. The near absence of contemporary research reflects ongoing data invisibility and structural factors affecting AI/AN young adults. AI/AN college students represent a critical yet overlooked population for catch-up HPV vaccination, requiring community-engaged strategies to advance cancer equity.

## 1. Introduction

Human papillomavirus (HPV) is the most prevalent sexually transmitted infection globally [1], with approximately 13 million new infections occurring each year in the United States alone [2]. While most HPV infections are transient, persistent infection with the high-risk HPV types 16 and 18 can lead to cancer in the cervix, vagina, vulva, oropharynx, rectum, anus, and penis [3]. American Indian and Alaska Native (AI/AN) communities bear a disproportionate burden of several HPV-associated cancers. Compared to non-Hispanic White women, AI/AN women experience higher cervical cancer incidence and mortality despite the availability of effective preventive tools such as HPV vaccination [4,5,6]. For example, in the Pacific Northwest, AI/AN women experienced approximately 1.5 times the cervical cancer incidence and 1.8 times the cervical cancer mortality of non-Hispanic White women [6]. These inequities position HPV vaccination as a critical cancer prevention strategy for AI/AN populations.

The HPV vaccine has been shown to significantly reduce the incidence of HPV associated cancers and is indicated for individuals between ages 9–45 years. Routine administration of the HPV vaccination is recommended for adolescents at ages 11–12 years, while catch-up vaccinations are recommended for all persons through age 26 years. In the United States, the HPV vaccine is provided at no cost through the Vaccines for Children (VFC) program to eligible children and adolescents through age 18, including American Indian and Alaska Native youth; young adults who have aged out of VFC eligibility, however, may face out-of-pocket costs for catch-up vaccination [7]. For adults ages 27–45 years, a shared clinical decision-making approach is recommended to identify if the individual will benefit from receiving the vaccination [8]. Despite the safety and efficacy of HPV vaccines in preventing HPV-related cancers [9,10], HPV vaccination rates in the United States remain below public health targets. In 2023, only 61.4% of adolescents aged 13–17 years were up to date on their HPV vaccinations [11], falling significantly below the Healthy People 2030 goal of 80% [12]. These shortfalls at younger ages have downstream consequences for young adults; an estimated 23 million emerging adults between the ages of 18 and 26 are unvaccinated [13], highlighting a missed opportunity for cancer prevention.

Emerging adulthood is a pivotal period for adopting preventive health behaviors, yet HPV vaccination efforts during this stage remain underprioritized in public health initiatives. Among general young adult and college populations, studies have documented suboptimal HPV vaccine initiation and completion and low levels of HPV knowledge, awareness, and risk perception [14]. College students frequently report misconceptions about HPV transmission and cancer risk, uncertainty about vaccine eligibility, and concerns about side effects or stigma associated with sexually transmitted infections [15]. These patterns suggest that substantial gaps in HPV-related knowledge and vaccine uptake exist even before considering the compounded effects of structural inequities among marginalized populations.

Because HPV vaccination is routinely administered in early adolescence, most existing research focuses on individuals younger than age 17 and their parents [16,17]. Although emerging adults are eligible for catch-up vaccinations, they are often overlooked, despite the fact that they may engage in higher-risk sexual behaviors that increase their susceptibility to HPV infection [18]. Unlike childhood vaccination, which is primarily guided by pediatricians and parental consent [15], college students are more likely to make independent health-related decisions [19]. College settings offer unique opportunities for intervention: campuses often have student health centers that can provide on-site vaccination, integrated electronic health records that facilitate reminders and series completion, and established communication channels such as orientation, residence halls, student organizations, and social media that can disseminate targeted health education at scale. The high concentration of young adults, combined with existing clinical and outreach infrastructure, makes college campuses a strategic venue for catch-up HPV vaccination and health promotion.

Within this broader landscape of underutilized HPV vaccination among emerging adults, AI/AN populations face additional and intersecting challenges. In 2019, HPV vaccination coverage for at least one dose was lower among AI/AN adolescents (71.1%) than among their Hispanic (76.8%), Asian (74.8%), multiracial (73.0%), and Black (72.0%) peers [20]. Nationally, HPV vaccination coverage has plateaued, showing no increase for the second consecutive year in 2023, a stagnation attributed in part to disruptions in preventive care during the COVID-19 pandemic [11]; contemporary estimates disaggregated specifically for AI/AN adolescents remain limited by small sample sizes and racial misclassification. HPV vaccination is not federally mandated in the United States, and only a few jurisdictions (e.g., Hawaii, Rhode Island, Virginia, and Washington, DC) require it for school entry [21]. AI/AN healthcare access is shaped by a complex system that includes the Indian Health Service (IHS), tribally operated facilities, urban Indian health programs, and non-tribal healthcare systems. These systems are often underfunded, geographically distant, and variably accessible based on tribal citizenship, insurance coverage, and residence [22]. Many AI/AN young adults attending college may be far from their home communities and usual sources of care, encounter fragmented coverage, or be ineligible for IHS services, creating additional barriers to vaccine access.

Furthermore, research suggests that historical trauma within AI/AN communities has contributed to mistrust in the medical system [22,23,24]. Concerns about exploitation, experimentation, and inadequate consultation have shaped perceptions of biomedical interventions, including vaccines [23]. When combined with structural barriers and data invisibility, these factors may compound the risk of under-vaccination and HPV-related cancer disparities for AI/AN emerging adults. Given the elevated burden of HPV-related cancers among AI/AN populations, the structural and historical context of healthcare access, and the unique opportunities for intervention within college settings, it is essential to examine HPV vaccination uptake specifically among AI/AN college students.

Consistent with JBI guidelines, a preliminary search was conducted on 1 March 2024, to identify existing scoping reviews and/or protocols (distinct from the six-database search of the primary literature described in the Methods). The search included the JBI Database of Systematic Reviews, MEDLINE (Ovid), Prospero, and Open Science Framework. No existing reviews or protocols that addressed this topic were identified confirming an explicit gap in the literature: to date, no synthesis has mapped evidence on HPV vaccination specifically among AI/AN college students. The rationale behind using a scoping review is that this method provides a systematic approach to map key concepts in an emerging field where evidence is limited and fragmented. This approach is particularly important given the intersection of two understudied groups, AI/AN populations and college students, and the lack of prior reviews addressing HPV vaccination in this context.

The objective of this scoping review is to map the current evidence on HPV vaccination among AI/AN college students aged 18–26, including vaccine uptake, knowledge, perceptions, and barriers. By mapping the existing literature, this review aims to highlight key factors influencing vaccine uptake, and to reveal gaps in the current evidence to inform future research. The questions that guided this scoping review were: (1) What is known about HPV vaccination uptake, knowledge, perceptions, and barriers among AI/AN college students aged 18–26? (2) What gaps exist in the current literature that limit understanding of HPV vaccination behaviors and decision-making within this population? The questions were developed using the JBI scoping review guidelines.

## 2. Material and Methods

This scoping review was conducted following the JBI approach for scoping reviews and the Preferred Reporting Items for Systematic Reviews and Meta-Analyses extension for Scoping reviews checklist (PRISMA-ScR) [25] [Supplementary Materials File S1]. Following input from all authors, a protocol was registered with the Open Science Framework on 9 July 2025 (https://osf.io/t68jg, (accessed on 26 July 2026)). This review was conducted in accordance with the aforementioned protocol.

An experienced health sciences librarian provided guidance on the development of the initial search strategy in August 2024. Following discussion with the team, this strategy was adapted for each of the databases that were searched: MEDLINE (Ovid), CINAHL Ultimate, Embase (Ovid), ERIC (Ovid), Scopus, and Web of Science Core Collection. The search strategies were developed and iteratively refined during 2024 (June and August iterations), and the finalized strategy was executed as the final search on 4–7 May 2025. No restrictions were placed on publication year. Appendix A provides detailed search strategies for each database, including controlled vocabulary terms, keywords, and the search functionality (e.g., truncation, Boolean operators, field-specific searches) utilized. Search results were limited to English-language literature; application of this limit revealed that the majority of results in each database were in English.

Eligible articles included college students aged 18–26 who were enrolled in a college or university setting and self-identified as AI/AN. Research studies were included if there were at least a portion of the participants who identified as AI/AN. In addition, articles included in this review reported on perceptions, barriers, and/or knowledge influencing HPV vaccine uptake. Studies that fell within the following categories were excluded: unpublished primary studies, quality improvement projects, theses/dissertations, reports from the gray literature, reviews, conference abstracts, and editorial/position papers. In addition, articles that examined college students without explicitly identifying AI/AN populations were excluded, as were those conducted in institutionalized settings (e.g., inpatient units) or outpatient settings (e.g., community clinics).

Citations were imported into Covidence, and duplicates were removed by the program. Titles and abstracts were screened for relevance using predetermined eligibility criteria, and the full texts of potentially relevant articles were retrieved and assessed for inclusion. To ensure consistency and completeness, two reviewers pilot-tested the extraction form in Covidence on three representative sources before proceeding with independent extraction. All levels of screening were performed independently by two reviewers, with conflicts resolved by a third reviewer. Data were extracted in duplicate using a standardized form in Covidence, and any discrepancies were resolved through discussion. Data extracted from included studies included study characteristics (e.g., citation, location, study objective, sample characteristics, and study approach) and HPV vaccination-related findings (e.g., knowledge and awareness, perceptions and attitudes, barriers to vaccination, and recommendations for intervention). Extracted data were summarized descriptively and synthesized narratively according to recurring themes.

## 3. Results

**Figure 1:**
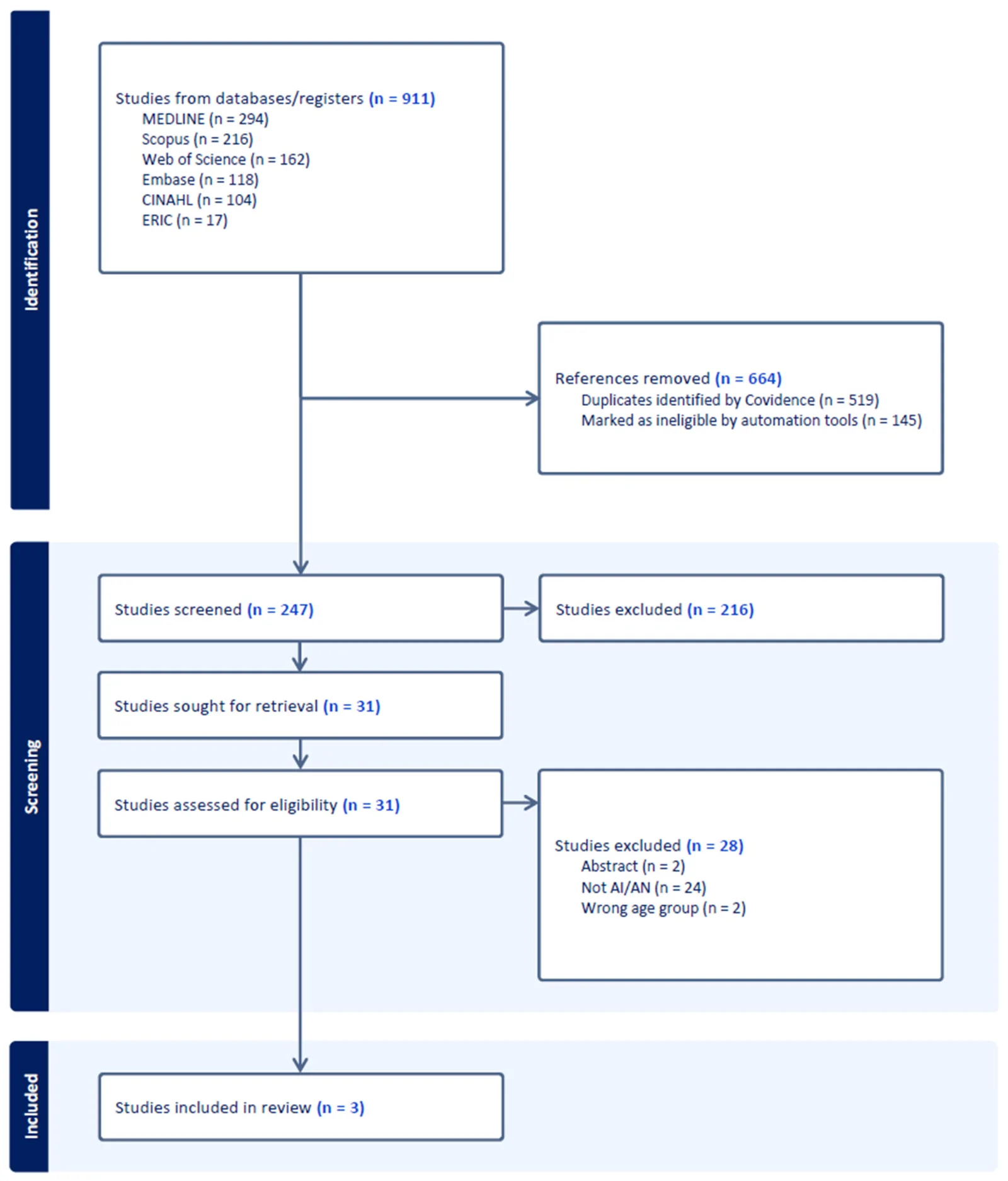
PRISMA diagram [25].

A search of electronic databases yielded a total of 911 citations. Covidence software (https://www.covidence.org/ (accessed on 9 July 2025)) was used by the researchers to assist with a systematic approach to screening, review, and extraction. A total of 519 duplicates were removed, and 145 were marked as ineligible by an automation tool in Covidence. The titles and abstracts of 247 articles were screened independently by two reviewers, and 216 papers with irrelevant titles and abstracts that did not meet the inclusion criteria were excluded. The remaining 31 full-text articles were assessed for eligibility. Discrepancies were resolved by a third reviewer. Ultimately, 28 full-text articles were excluded as they were not directly relevant to the research questions guiding this scoping review. Of these, 24 did not meet the population inclusion criteria. In 21 of those studies, AI/AN participants were either misclassified, not disaggregated from broader racial or ethnic groups, or combined with other populations, which limited the ability to draw meaningful, population-specific conclusions. Two additional studies did not exclusively examine individuals aged 18 to 26, and two were conference abstracts without a corresponding full-text publication. A summary of the search and selection process is presented in the PRISMA chart (Figure 1).

The results of this comprehensive search demonstrate that there is a paucity of research specifically examining the perceptions, knowledge, and barriers related to HPV vaccine uptake among AI/AN college students. A total of three articles met the inclusion criteria. These three articles were from the same research study using data collected in 2009, but each article reported on different aspects of the study data set and results.

### 3.1. Study Characteristics

The three publications meeting inclusion criteria were derived from a single mixed-methods, cross-sectional study conducted in 2009 in the United States. The study focused on AI college students and used both surveys and focus group interviews to explore HPV vaccine-related knowledge, perceptions, and barriers. While all three articles were based on the same dataset and methodological approach, each publication emphasized different participant groups (genders) and analytic priorities.

The articles (*N* = 3) were published between 2011 and 2016. Samuel-Nakamura and Hodge [26] focused exclusively on female participants and emphasized qualitative findings to explore cultural themes related to HPV vaccine attitudes. Hodge focused exclusively on male participants, using both survey and focus group data to examine gender-specific perceptions of vaccine acceptability [27]. Hodge et al. included both male and female participants and took a broader view, reporting quantitative findings related to HPV knowledge and general awareness [28].

All participants were AI college students attending two West Coast and two Southwestern colleges in the United States. The included publications did not report the number of tribes or the specific tribal affiliations represented among participants. Each article drew from the same original participant pool and data sources but reported distinct thematic and subgroup findings. Common topics across the three publications included low levels of HPV knowledge and awareness, vaccine perceptions and attitudes, and barriers to vaccination. Table 1 provides a summary of the included studies.

### 3.2. Factors Influencing Uptake

#### 3.2.1. Knowledge and Awareness

Low levels of HPV-related knowledge were consistently identified as a major barrier to vaccine uptake among AI college students. Quantitative data reflected the knowledge gap: on an 8-point Likert scale, the mean score for males was 2.22 and for females 2.79, indicating poor understanding of HPV [26]. This is further supported by the qualitative data in all three publications. A gap in male knowledge and awareness was evident, with one male participant stating, “I’ve never heard of it until now,” and others indicating they were not aware of anyone who received the HPV vaccine [27]. Similarly, female participants were also reported to have scant knowledge about the HPV vaccine [26]. One female participant indicated that they believed the low level of knowledge was due to the lack of education on the reservation [26]. In both the male and female focus groups, participants expressed limited understanding of HPV transmission, consequences, and vaccine availability [26].

#### 3.2.2. Perceptions and Attitudes

Many students did not perceive themselves to be at risk for HPV, which affected their willingness to engage in preventative behaviors. Among males, there was a prevalent belief that HPV primarily affects women, with nearly half of them (52%) indicating they did not worry about HPV because they were male [28]. This low perceived relevance and responsibility was made visible by one participant’s statement, “I guess (HPV programs are designed) in particular for women because it seems they are the ones that are affected the most by this” [27]. Although female students scored 1.5 times higher than males on the HPV attitude scale, indicating a more favorable view of the HPV vaccine, they still demonstrated a low-risk perception and regarded vaccination as having little importance [26].

In addition, participants reported that social and cultural influences shaped attitudes toward vaccination. When cultural values and traditional beliefs were considered, participants noted that discussions of sexual health were often considered taboo in their culture, which created discomfort and reluctance in discussing sexually related topics such as the vaccine [26,27]. Male students expressed that traditional Native American beliefs influenced by religion often prohibited open discussion surrounding sexuality, especially among close family members [27]. One participant noted that his parents believed sexual education should be the responsibility of the school system rather than the parents [27]. Female participants also emphasized the role of culture in health and healthcare decisions, voicing their reliance on traditional medicine for healing [26]. One student shared, “There probably is a cure, I mean from a traditional perspective, not White man’s medicine’’ [28].

#### 3.2.3. Barriers to Vaccination

Participants identified a range of barriers that hindered HPV vaccine uptake, including issues related to accessibility and mistrust stemming from historical trauma. Perceived barriers to vaccination were relatively low for both groups, with males scoring a 0.83 and females scoring a 1.00 on a 0–4 scale. However, females consistently reported higher rates for specific barriers, including uncertainty about where to seek HPV vaccine information (32.35% vs. 26.09%) and discomfort in seeking such information (6.06% vs. 4.35%) [28]. In addition, the qualitative data revealed more nuanced challenges that influenced decision-making [26,27,28].

Within the focus groups, female students discussed issues related to structural accessibility, including limited access to healthcare services. This particularly affected those living in rural communities and/or on a reservation, with one participant explaining, “I lived an hour away … so just going back three times, that whole series, I couldn’t do it” [26,28]. Others emphasized that not all AI’s have insurance or have access to Indian Health Service (IHS) clinics, which serves as a barrier to receiving the vaccine [26]. While issues related to access emerged in the female-focused publication [26], no direct mention of access or other structural barriers, such as transportation, insurance, or clinical availability, was noted in the male-focused article [27].

Historical trauma and intergenerational mistrust of healthcare systems further influenced how participants viewed vaccination, particularly when it was perceived as being promoted by the government. Participants referenced past historical events, such as forced sterilizations [26], as part of the reason for skepticism toward vaccination campaigns. One student remarked, “For Natives, vaccinations are crazy. The whole history with the IHS, I’m not sure people believe they can trust them” [28]. Feelings of mistrust about pharmaceutical companies and physicians were also evident, with one participant questioning, “Why are American Indian women just being targeted? Is it to decrease the population or what are the side effects?” [26,28].

### 3.3. Recommendations for Interventions

All three publications provided practical strategies to address the low HPV-related knowledge, limited perceived risk, and cultural barriers influencing vaccination among AI college students. Samuel-Nakamura and Hodge reported that cultural stigma around sexual health was a recurring barrier in the theme “We don’t talk about it”, highlighting the importance of discreet, culturally appropriate messaging [26]. Culturally tailored education that integrates traditional values, reduces stigma, and aligns with community communication preferences emerged as a priority [26,27,28]. Engaging families, communities, schools, and clinic personnel was viewed as an important first step in developing and implementing educational and immunization initiatives [26]. Both male and female students identified schools, campus health centers, and trusted healthcare providers as preferred venues for receiving HPV information, underscoring the importance of delivering content in culturally appropriate settings.

Participants also highlighted the role of provider engagement. Students were more likely to consider vaccination when healthcare professionals offered clear, proactive, culturally appropriate recommendations. Addressing misinformation about side effects and clarifying the vaccine’s benefits for both men and women may improve acceptability, particularly among male students who frequently perceived HPV as a women’s health issue [27]. As one student explained, “I would not recommend anything unless I knew a lot about it,” underscoring the need for accurate, accessible HPV information [26]. Finally, integrating prevention messages that also address other HPV-related cancers and genital warts, conditions affecting both sexes, may increase perceived relevance and encourage shared responsibility for prevention.

## 4. Discussion

This scoping review of the literature reveals a significant gap in research examining the HVP vaccine perception, knowledge, and barriers among AI/AN college students. From over 900 articles screened, only three publications from a single study in 2009 met the inclusion criteria. This underscores the need for more up-to-date, population-specific research to inform culturally sensitive interventions for this population. Findings from the three included manuscripts highlight low HPV-related knowledge, limited perceived risk, cultural stigma around discussing sexual health, mistrust in the healthcare system rooted in historical trauma, and structural access issues impacting HPV vaccine uptake among AI/AN college students.

AI/AN college students represent a unique subgroup of emerging adults who are eligible for catch-up HPV vaccination while increasingly assuming responsibility for their own healthcare decisions. Despite this critical developmental stage, contemporary evidence describing HPV vaccination among this population remains extremely limited. The absence of current research restricts our understanding of how emerging adults navigate vaccination decisions within the context of college, cultural identity, and evolving healthcare access.

The limited research on this topic is itself a critical finding, particularly given that 21 studies were excluded because AI/AN participants were misclassified, not disaggregated from other racial or ethnic groups, or aggregated with heterogeneous populations. These data collection and reporting practices limit the development of population-specific conclusions and perpetuate health disparities. Additionally, there have been substantial updates to HPV vaccination recommendations since these data were collected in 2009. For example, in 2011, routine vaccination recommendations were expanded to include boys, and current guidance recommends routine vaccination for all children aged 11–12 and catch-up immunization through age 26 years [29]. These changes in recommendations limit our understanding of current beliefs, barriers, and facilitators of HPV vaccination in AI/AN college students.

The findings of this review align with the broader literature documenting low-HPV-related knowledge, suboptimal vaccine uptake, and misperceptions among general college student populations [14]. Similar to non-AI/AN young adults, AI college students in the included studies demonstrated limited understanding of HPV transmission, cancer risk, and vaccine benefits, and often perceived HPV primarily as a women’s health issue. However, the AI-specific findings contextualize these gaps within cultural norms, structural access barriers, and historical experiences with healthcare systems, underscoring the need for interventions that are not only age-appropriate but also culturally grounded and attentive to AI/AN healthcare realities.

The barriers identified in this review are echoed in HPV vaccination research across a range of international and cross-cultural settings, situating the AI/AN experience within a broader global pattern. Systematic reviews from sub-Saharan Africa have documented how stigma, cost, misinformation, and weak health-system infrastructure suppress uptake [30], while a scoping review of Islamic countries found that religious concerns—including beliefs that the vaccine conflicts with religious norms or promotes promiscuity—shape acceptance [31]. Comparable sexual-health taboos and gendered decision-making appear among Mexican American adults, for whom cultural taboos and the framing of HPV as a women’s issue constrain vaccination [32], and across low-resource settings, where promiscuity concerns, religious beliefs, and gender norms recur as sociocultural barriers [33]. A recent systematic review likewise concluded that cultural norms and patriarchal family structures predominantly shape parental HPV vaccination decisions worldwide [34]. That these themes—sexual-health stigma, mistrust, gendered risk perception, and structural access barriers—parallel those observed among AI/AN college students underscores their cross-cultural salience while reinforcing the need for interventions tailored to the specific cultural and historical context of AI/AN communities.

### 4.1. Mistrust of Healthcare Systems and Historical Context

Qualitative findings across the included publications highlight how mistrust of healthcare systems, including IHS and other governmental institutions, shapes HPV vaccine decision-making among AI college students. Participants referenced historical events such as forced sterilizations and expressed concerns about being “targeted” by vaccination campaigns, questioning the motives of healthcare institutions and pharmaceutical companies [26,27,28]. These narratives are consistent with broader evidence that historical trauma, unethical research practices, and systemic discrimination have fostered deep skepticism toward biomedical interventions within AI/AN communities [23].

Because the three included publications all derive from a single 2009 mixed-methods study, the mistrust theme reflects that one study’s data as reported across its publications rather than a pattern replicated across independent studies. The strength of our conclusion about mistrust therefore derives from integrating these qualitative findings with extensive literature documenting AI/AN communities’ historical and contemporary experiences with healthcare systems. Taken together, these sources underscore that efforts to increase HPV vaccination among AI/AN college students must prioritize trust-building through meaningful community engagement, respect for tribal sovereignty, transparent communication, and adherence to Indigenous data governance principles.

### 4.2. Implications for Practice and Education

Given the consistently low levels of knowledge regarding HPV, HPV vaccination, and the link between HPV and cancer among college students [14,35,36], educational interventions remain a priority. Healthcare providers working with and caring for AI/AN college students must utilize culturally sensitive approaches that acknowledge current barriers and historical trauma related to vaccine uptake. Based on the finding that sexual health discussions are frequently viewed as taboo within AI communities [26,27,28], health education strategies that provide essential health information, while respecting cultural values, must be implemented [37].

The gender differences in views on HPV risk should also be considered. Educational materials should emphasize that HPV impacts all genders and highlight the role of HPV vaccination in preventing genital warts and various cancers [3]. Among general college populations, interventions that address affective responses, perceived norms, and decision-making processes have shown promise for improving HPV vaccine uptake [19,38,39]. For AI/AN college students, these strategies should be adapted to incorporate Indigenous ways of knowing, community narratives about cancer and survivorship, and strengths-based framing that emphasizes cultural resilience.

Educational interventions should be developed in collaboration with AI/AN communities to ensure that these materials are both culturally relevant and appropriate [40]. With both male and female students identifying campus health centers as preferred venues for receiving HPV vaccine-related information, these settings should be prioritized when developing and implementing interventions. Campus-based programs can leverage existing student support structures, such as Native American student centers, cultural organizations, and peer mentors, to co-create messaging and outreach strategies. These targeted educational interventions should acknowledge and respect traditional healing practices while discussing HPV vaccination as a complementary prevention strategy.

Healthcare providers must also be aware of structural barriers that impact uptake of HPV vaccination, including the ability to complete a multi-dose series, access to transportation, and the ability to afford preventative healthcare. Campus health centers are in a unique position to mitigate these barriers by offering vaccinations on-site, extending clinic hours, coordinating vaccine delivery across academic terms, and offering programs that can assist or fully cover the cost of vaccines. Additional strategies, such as reminder systems and text message campaigns, could improve completion rates of HPV vaccination series [41]. Coordinating care between campus health services, IHS or tribally operated facilities, and community clinics may help ensure continuity of vaccination for students who travel between home and school.

### 4.3. Implications for Health Policy

The lack of disaggregated data on AI/AN populations revealed through this review brings to light systematic data collection failures that perpetuate health disparities. In 2024, the Office of Management and Budget revised Statistical Policy Directive No. 15 to require that race and ethnicity be collected in a single combined question and that detailed categories be included by default. Health researchers must incorporate these standards when developing, collecting, and reporting demographic data in their studies. In addition to federal agencies requiring compliance with Statistical Policy Directive 15, institutional review boards and journal editors should enforce these standards as a condition of study approval and publication. This is a step in addressing the health inequities among understudied, underrepresented, and underreported populations [42].

Structural barriers identified through this review, including geographic barriers to care, limited access to IHS facilities, and lack of insurance coverage, require comprehensive policy solutions. Increasing funding to IHS to expand coverage for preventative healthcare for eligible AI/AN college students, supporting tribally driven vaccination clinics for rural and reservation communities, could address critical accessibility challenges. Complementary policies should ensure that HPV vaccination is covered without cost-sharing through campus-based health plans, Medicaid, and other payers so that AI/AN students who obtain care outside IHS systems are not financially disadvantaged.

### 4.4. Implications for Future Research

The limited availability of data related to this topic, dating back over 14 years, highlights an urgent need for current research examining HPV vaccine uptake among AI college students. Large-scale, mixed-methods studies are needed to generate contemporary evidence on HPV vaccine uptake, knowledge, perceptions, and barriers among this population. Future research should also explore how updates to the HPV vaccine recommendations, including routine vaccination for males, the role of technology and social media, and the COVID-19 pandemic and vaccines, have influenced HPV vaccine knowledge, attitude, and uptake in this population. Longitudinal studies that track HPV vaccine initiation and completion could identify further barriers and opportunities for intervention among AI college students.

Additionally, studies should investigate the differences between rural, urban, and reservation-dwelling AI/AN college students, as cultural connections and access to healthcare may significantly vary [40]. Future research should also examine the role that technology and social media play in HPV vaccine promotion and education among AI/AN college students. Digital interventions have the potential to provide information that is accessible and culturally tailored, while respecting privacy concerns related to sexual health topics. Findings from this review suggest that there are gender differences in knowledge and attitudes related to HPV vaccine uptake. Future research should understand how Two-Spirit and LGBTQ+ AI/AN young adults navigate decisions related to HPV vaccination within their cultural and college communities.

With 574 federally recognized American Indian tribes and Alaska Native villages in the United States, it is essential to consider the distinct beliefs and cultural practices of each tribe when designing future studies and culturally sensitive interventions. A community-based participatory research (CBPR) approach that involves community members, in this case, AI/AN college students, in the research process, has the potential to create meaningful and impactful interventions that are tailored to this population. LaVeaux and Christopher outlined nine recommendations for researchers who are interested in engaging in CBPR with tribal communities [43]. Key recommendations related to this work include acknowledging historical experiences with research and working to overcome research’s negative image, recognizing tribal sovereignty, understanding tribal diversity and its implications, interpreting data within a cultural context, and utilizing Indigenous ways of knowing [43].

On a broader level, studies evaluating the effectiveness of single-dose HPV vaccine schedules and how this impacts uptake of AI/AN college students and the young adult community at large should be conducted. In 2022, the World Health Organization updated dosing recommendations for HPV vaccination to include an alternative single-dose schedule. This was based on evidence that a single-dose HPV schedule provides similar protection as a multidose schedule against both initial and persistent HPV infection [44].

### 4.5. Strengths and Limitations

This review has several strengths and limitations. A key strength is the use of established methodological guidance for scoping reviews, including the JBI approach and the PRISMA-ScR checklist [25]. The protocol was registered a priori on the Open Science Framework, enhancing transparency and methodological rigor. A health sciences librarian partnered with the research team to design and refine comprehensive search strategies across multiple databases, and all screening and data extraction were conducted independently by at least two reviewers, with adjudication by a third reviewer when needed. These processes strengthen the credibility and reproducibility of the review. Another important strength is the explicit focus on AI/AN college students and the insistence on disaggregated data, which addresses a critical gap in the HPV vaccination literature.

This review is limited by the scarcity of available research meeting inclusion criteria, with only three eligible publications, all derived from a single mixed-methods study conducted in 2009. As such, the evidence may not reflect current HPV vaccination rates, awareness, or attitudes among AI college students. The reliance on one 2009 dataset is an equity concern, as this review illustrates systemic neglect of AI/AN college students in vaccine research for over a decade. The geographic scope was narrow, encompassing only four institutions on the West Coast and the Southwestern United States, which may not represent the diversity of AI student experiences’ nationally. Additionally, the original study did not include students identifying as Alaska Native, limiting the generalizability. Many potentially relevant studies were excluded due to racial misclassification or aggregation of AI/AN participants with other groups, precluding population-specific analyses. Additionally, the focus on the higher education-specific settings omits perspectives from other healthcare contexts where vaccination may occur. Collectively, these limitations underscore the urgent need for updated, geographically diverse, and methodologically rigorous research to inform targeted interventions for this underserved population.

## 5. Conclusions

This scoping review revealed a lack of research specifically examining the knowledge, perceptions, and barriers related to HPV vaccine uptake among AI/AN college students. These findings are of particular importance because this population is eligible for catch-up vaccination yet frequently overlooked, despite evidence of higher-risk sexual behaviors that may increase susceptibility to HPV infection [18]. Unlike childhood vaccination, which is largely directed by pediatricians and parental consent [15,45], college students can exercise greater autonomy over their healthcare decisions and may have access to campus-based health services [19]. These factors position AI/AN college students within a unique and opportune stage for intervention.

Findings from this review indicate that AI college students experience low HPV-related knowledge, limited perceived risk, cultural stigma surrounding discussions of sexual health, structural access barriers, and concerns about healthcare systems, including IHS. These concerns echo a broader body of work documenting how historical and ongoing injustices in research and healthcare have contributed to mistrust among AI/AN communities [22,23,24]. Together, the evidence suggests that efforts to improve HPV vaccination among AI/AN college students must directly address data invisibility, structural inequities, and trust in healthcare systems, rather than focusing solely on individual-level education.

## Figures and Tables

**Table 1:** Summary of reviewed articles.

Citation	Location	Study Objective	Sample/Participants	Study Approach	Findings
Hodge et al. (2011) [28]	Four large colleges/universities, 2 in California and 2 in Arizona	To examine HPV vaccine readiness and explore knowledge, attitudes, risk perceptions, and decision-making among American Indian (AI) college students aged 18–26 years.	AI college students *N* = 57 (34 female, 23 male)	Mixed-methods study Cross sectional Qualitative: Focus groups Quantitative: Survey	Significant sex differences in HPV risk perception (*p* = 0.008). Females: higher knowledge and perceived risk, but less likely to recommend the vaccine, and lacked knowledge on information sources. Males: 52% perceived themselves at low risk. Six influencing factors: limited knowledge, access barriers, fear of side effects, cultural influences, adverse historical experiences, and poor risk perception.
Hodge (2014) [27]	Four large colleges/universities, 2 in California and 2 in Arizona	To explore American Indian (AI) male college students’ attitudes, knowledge, and perceptions of HPV prevention and transmission, and identify cultural and educational barriers to HPV education.	AI college students Reported AI male college students *N* = 23 (male subsample of *N* = 57)	Mixed-methods study Cross sectional Qualitative: Focus groups Quantitative: Survey	AI males reported little/no responsibility for HPV prevention (*p* = 0.048) and poor personal risk perception (*p* = 0.0001). Low HPV knowledge (*p* = 0.003) and lenient attitudes reflected disregard for safer sexual practices. Misinformation about HPV vaccination reduced intention to change behaviors. Identified cultural barriers and preferences for HPV information sources.
Samuel-Nakamura & Hodge (2016) [26]	Two West Coast and two Southwestern U.S. colleges	To assess HPV-vaccine readiness among American Indian women college students and identify modifiable and non-modifiable factors influencing their vaccine decision-making.	AI college students Reported on AI female college students *N* = 34 (female subsample of *N* = 57)	Mixed-methods study Cross sectional Qualitative: Focus groups Quantitative: Survey	AI women demonstrated mixed HPV vaccine readiness, influenced by both modifiable and non-modifiable factors. Barriers included low knowledge, limited access, fear of side effects, and cultural stigma. Non-modifiable influences such as historical trauma and community norms shaped decision-making. Family, community, and clinical engagement were emphasized as first steps in supporting education and vaccination.

## Data Availability

No new data were created or analyzed in this study. Data sharing is not applicable to this article.

## Supplementary Materials

The following supporting information can be downloaded at https://www.mdpi.com/article/10.3390/ijerph23081027/s1, File S1: Preferred Reporting Items for Systematic reviews and Meta-Analyses extension for Scoping Reviews (PRISMA-ScR) Checklist.

## Appendix A. Electronic Database Search Strategies

**Table A1:** Medline (Ovid) and CINAHL (EBSCO)—initial search strategy (developed 3 June 2024).

Search	Query	Results
#1	exp Papillomavirus Vaccines	10,665
#2	((papilloma$ or hpv) adj2 (vaccin$ or immuniz$)).mp.	15,665
#3	(gardasil$ or ceravix$ or cervarix$ or silgard$).mp.	765
#4	or/1–3	15,715
#5	Universities/	54,844
#6	(universit$ or college$).mp.	653,668
#7	5 or 6	653,668
#8	4 and 7	956
#9	l/8 lg = en	934
#10	remove duplicates from 9	934
#11	exp “American Indian or Alaska Native”/	23,644
#12	(native american$ or american indian$ or amerind$).mp.	18,000
#13	(aian or ai an).mp.	1624
#14	(indigenous$ or tribe or tribes or tribal$).mp.	63,399
#15	or/11–14	89,899
#16	10 and 15	7
#17	10 not 16	927
#18	(universit$ or college$ or student$ or campus$).ti,hw,kf,jw.	530,563
#19	17 and 18	475

Limits: Publication dates of 1946 and later; English language. $ is a part of the actual search strategy.

**Table A2:** Medline (Ovid) and CINAHL (EBSCO) search strategy—finalized search strategy (developed 8 August 2024; executed as final search 4–7 May 2025).

Search	Query	Results
#1	exp Papillomavirus Vaccines/(10,773)	10,773
#2	((papilloma$ or hpv) adj2 (vaccin$ or immuniz$)).mp.	15,870
#3	(gardasil$ or ceravix$ or cervarix$ or silgard$).mp.	769
#4	or/1–3	15,921
#5	Universities/	55,450
#6	(universit$ or college$ or undergrad$).mp.	713,648
#7	((grad$ or doctoral$ or phd or ph d or master$) adj1 student$).mp.	14,398
#8	or/5–7	723,374
#9	4 and 8	1029
#10	l/9 lg = en (1007)	1007
#11	remove duplicates from 10	1005
#12	exp “American Indian or Alaska Native”/	23,760
#13	(native american$ or american indian$ or amerind$).mp.	18,236
#14	(aian or ai an).mp.	1663
#15	(indigenous$ or first nation$ or tribe or tribes or tribal$ or cherokee$ or choctaw$ or chickasaw$ or creek$ or seminole$ or muscogee$).mp.	74,269
#16	exp Racial Groups/	118,816
#17	exp Ethnicity/	111,655
#18	“Ethnic and Racial Minorities”/	1068
#19	(race or races or racial$ or racis$ or ethnic$ or minorit$).mp.	460,175
#20	or/12–19	603,951
#21	11 and 20	145
#22	l/21 lg = en	145
#23	remove duplicates from 22	145

Limits: Publication dates of 1946 and later; English language. $ is a part of the actual search strategy.

## References

[1] World Health Organization (2024). Human Papillomavirus and Cancer [Fact Sheet]. https://www.who.int/news-room/fact-sheets/detail/human-papilloma-virus-and-cancer.

[2] Centers for Disease Control and Prevention (2024). About Human Papillomavirus (HPV). https://www.cdc.gov/hpv/about/index.html.

[3] National Cancer Institute (2025). HPV and Cancer. https://www.cancer.gov/about-cancer/causes-prevention/risk/infectious-agents/hpv-and-cancer.

[4] Melkonian S.C., Henley S.J., Senkomago V., Thomas C.C., Jim M.A., Apostolou A., Saraiya M. (2020). Cancers Associated with Human Papillomavirus in American Indian and Alaska Native Populations—United States, 2013–2017. MMWR Morb. Mortal. Wkly. Rep..

[5] Bruegl A.S., Emerson J., Tirumala K. (2023). Persistent disparities of cervical cancer among American Indians/Alaska natives: Are we maximizing prevention tools?. Gynecol. Oncol..

[6] Bruegl A.S., Joshi S., Batman S., Weisenberger M., Munro E., Becker T. (2020). Gynecologic cancer incidence and mortality among American Indian/Alaska Native women in the Pacific Northwest, 1996–2016. Gynecol. Oncol..

[7] Centers for Disease Control and Prevention (2025). Vaccines for Children (VFC) Program: Program Eligibility. https://www.cdc.gov/vaccines-for-children/hcp/program-eligibility/index.html.

[8] Meites E., Szilagyi P.G., Chesson H.W., Unger E.R., Romero J.R., Markowitz L.E. (2019). Human Papillomavirus Vaccination for Adults: Updated Recommendations of the Advisory Committee on Immunization Practices. MMWR Morb. Mortal. Wkly. Rep..

[9] Kamolratanakul S., Pitisuttithum P. (2021). Human Papillomavirus Vaccine Efficacy and Effectiveness against Cancer. Vaccines.

[10] Waheed D.E., Schiller J., Stanley M., Franco E.L., Poljak M., Kjaer S.K., Del Pino M., van der Klis F., Schim van der Loeff M.F., Baay M. (2021). Human papillomavirus vaccination in adults: Impact, opportunities and challenges—A meeting report. BMC Proc..

[11] Pingali C., Yankey D., Chen M., Elam-Evans L.D., Markowitz L.E., DeSisto C.L., Schillie S.F., Hughes M., Valier M.R., Stokley S. (2024). National Vaccination Coverage Among Adolescents Aged 13–17 Years—National Immunization Survey-Teen, United States, 2023. MMWR Morb. Mortal. Wkly. Rep..

[12] National Center for Health Statistics (2024). Increase the Proportion of Adolescents Who Get Recommended Doses of the Human Papillomavirus (HPV) Vaccine (IID-08) [Data Summary]. Healthy People 2030. https://health.gov/healthypeople/objectives-and-data/browse-objectives/vaccination/increase-proportion-adolescents-who-get-recommended-doses-hpv-vaccine-iid-08.

[13] Williams W.W., Lu P.J., O’Halloran A., Kim D.K., Grohskopf L.A., Pilishvili T., Skoff T.H., Nelson N.P., Harpaz R., Markowitz L.E. (2017). Surveillance of Vaccination Coverage among Adult Populations—United States, 2015. MMWR Surveill. Summ..

[14] Albright A.E., Allen R.S. (2018). HPV Misconceptions Among College Students: The Role of Health Literacy. J. Community Health.

[15] Kasymova S., Harrison S.E., Pascal C. (2019). Knowledge and Awareness of Human Papillomavirus Among College Students in South Carolina. Infect. Dis..

[16] Radisic G., Chapman J., Flight I., Wilson C. (2017). Factors associated with parents’ attitudes to the HPV vaccination of their adolescent sons: A systematic review. Prev. Med..

[17] Hamzehgardashi Z., Keshavarz Z., Fakhri M., Nasiri M., Zeynab Hoseinnezhad S., Shasti L. (2022). A Systematic Review on Interventions Affecting Parents’ Knowledge and Attitudes about HPV Vaccine. Tabari Biomed. Stud. Res. J..

[18] Lee M., Gerend M.A., Whittington K.D., Collins S.K., McKinney S.L., Franca M.C., Boyer V.E., McKinnies R.C., Chen C.C., Villegas J. (2024). Factors associated with HPV-associated sexual risk behaviors among sexually active college students. J. Behav. Med..

[19] Olusanya O.A., Tomar A., Thomas J., Alonge K., Wigfall L.T. (2023). Application of the theoretical domains framework to identify factors influencing catch-up HPV vaccinations among male college students in the United States: A review of evidence and recommendations. Vaccine.

[20] Elam-Evans L.D., Yankey D., Singleton J.A., Sterrett N., Markowitz L.E., Williams C.L., Fredua B., McNamara L., Stokley S. (2020). National, Regional, State, and Selected Local Area Vaccination Coverage Among Adolescents Aged 13–17 Years—United States, 2019. MMWR Morb. Mortal. Wkly. Rep..

[21] Kaiser Family Foundation (2026). The HPV Vaccine: Access and Use in the U.S. https://www.kff.org/womens-health-policy/the-hpv-vaccine-access-and-use-in-the-u-s/.

[22] Bordeaux S.J., Baca A.W., Begay R.L., Gachupin F.C., Caporaso J.G., Herbst-Kralovetz M.M., Lee N.R. (2021). Designing Inclusive HPV Cancer Vaccines and Increasing Uptake among Native Americans-A Cultural Perspective Review. Curr. Oncol..

[23] Baker H.A., Klunk A., Calac A.J., Livermont T., Bell S. (2023). Improving Indian Health Service Vaccination Campaigns Across the Full Spectrum of Age, Clinical, and Public Health Settings. Am. J. Public Health.

[24] Gopalani S.V., Sedani A.E., Janitz A.E., Clifton S.C., Peck J.D., Comiford A., Campbell J.E. (2022). Barriers and Factors Associated with HPV Vaccination Among American Indians and Alaska Natives: A Systematic Review. J. Community Health.

[25] Tricco A.C., Lillie E., Zarin W., O’Brien K.K., Colquhoun H., Levac D., Moher D., Peters M.D.J., Horsley T., Weeks L. (2018). PRISMA Extension for Scoping Reviews (PRISMA-ScR): Checklist and Explanation. Ann. Intern. Med..

[26] Samuel-Nakamura C., Hodge F.S. (2016). Modifiable and Non-Modifiable Factors Associated with HPV Vaccine Decision-Making among American Indian Women College Students. Am. Indian Cult. Res. J..

[27] Hodge F.S. (2014). American Indian Male College Students Perception and Knowledge of Human Papillomavirus (HPV). J. Vaccines Vaccin..

[28] Hodge F.S., Itty T., Cardoza B., Samuel-Nakamura C. (2011). HPV vaccine readiness among American Indian college students. Ethn. Dis..

[29] Gargano J.W., Stefanos R., Dahl R.M., Castilho J.L., Bostick E.A., Niccolai L.M., Park I.U., Blankenship S., Brackney M.M., Chan K. (2025). Trends in Cervical Precancers Identified Through Population-Based Surveillance—Human Papillomavirus Vaccine Impact Monitoring Project, Five Sites, United States, 2008–2022. MMWR Morb. Mortal. Wkly. Rep..

[30] Kutz J.M., Rausche P., Gheit T., Puradiredja D.I., Fusco D. (2023). Barriers and facilitators of HPV vaccination in sub-saharan Africa: A systematic review. BMC Public Health.

[31] Kisa S., Kisa A. (2024). Religious beliefs and practices toward HPV vaccine acceptance in Islamic countries: A scoping review. PLoS ONE.

[32] Morales-Campos D.Y., Snipes S.A., Villarreal E.K., Crocker L.C., Guerrero A., Fernandez M.E. (2021). Cervical cancer, human papillomavirus (HPV), and HPV vaccination: Exploring gendered perspectives, knowledge, attitudes, and cultural taboos among Mexican American adults. Ethn. Health.

[33] Xu M.A., Choi J., Capasso A., DiClemente R.J. (2024). Improving HPV Vaccination Uptake Among Adolescents in Low Resource Settings: Sociocultural and Socioeconomic Barriers and Facilitators. Adolesc. Health Med. Ther..

[34] Salleh N.S., Abdullah K.L., Chow H.Y. (2025). Cultural barriers and facilitators of the parents for human papillomavirus (HPV) vaccination uptake by their daughters: A systematic review. J. Pediatr..

[35] Franca M.C., Boyer V.E., Gerend M.A., Lee M., Whittington K.D., McKinney S.L., Collins S.K., McKinnies R.C., Adjei Boakye E. (2023). College Students’ Awareness of the Link Between Human Papillomavirus (HPV) and HPV-Associated Cancers. J. Cancer Educ..

[36] Malik S., Mock K.O., Martillotti R., Caravella G., Zhou X., Mbamelu M., Scarbrough K.H. (2024). HPV Vaccines Among University Students: Understanding Barriers and Facilitators of Vaccine Uptake. Vaccines.

[37] Hodge F.S., Line-Itty T., Ellenwood C. (2014). Communication Pathways. Californian J. Health Promot..

[38] Barnard M., George P., Perryman M.L., Wolff L.A. (2017). Human papillomavirus (HPV) vaccine knowledge, attitudes, and uptake in college students: Implications from the Precaution Adoption Process Model. PLoS ONE.

[39] Harper K., Short M.B., Bistricky S., Kusters I.S. (2023). 1-2-3! Catch-Up for HPV: A Theoretically Informed Pilot Intervention to Increase HPV Vaccine Uptake among Young Adults. Am. J. Health Educ..

[40] Walters K.L., Johnson-Jennings M., Stroud S., Rasmus S., Charles B., John S., Allen J., Kaholokula J.K., Look M.A., de Silva M. (2020). Growing from Our Roots: Strategies for Developing Culturally Grounded Health Promotion Interventions in American Indian, Alaska Native, and Native Hawaiian Communities. Prev. Sci..

[41] Louw G.E., Hohlfeld A.S., Kalan R., Engel M.E. (2024). Mobile Phone Text Message Reminders to Improve Vaccination Uptake: A Systematic Review and Meta-Analysis. Vaccines.

[42] National Institutes of Health (2025). New Funding Opportunity Supports Research on Understudied, Underrepresented, and Underreported (U3) Populations of Women. https://orwh.od.nih.gov/in-the-spotlight/all-articles/new-funding-opportunity-supports-research-on-understudied-underrepresented-and-underreported-u3.

[43] Laveaux D., Christopher S. (2009). Contextualizing Cbpr: Key Principles of Cbpr Meet the Indigenous Research Context. Pimatisiwin.

[44] World Health Organization (2022). WHO Updates Recommendations on HPV Vaccination Schedule. https://www.who.int/news/item/20-12-2022-WHO-updates-recommendations-on-HPV-vaccination-schedule.

[45] Klasko-Foster L.B., Przybyla S., Orom H., Gage-Bouchard E., Kiviniemi M.T. (2020). The influence of affect on HPV vaccine decision making in an HPV vaccine naive college student population. Prev. Med. Rep..

